# Microbial containment device: A platform for comprehensive analysis of microbial metabolism without sample preparation

**DOI:** 10.3389/fmicb.2022.958785

**Published:** 2022-09-13

**Authors:** Mehdi Mohammadi, Stephanie L. Bishop, Raied Aburashed, Saad Luqman, Ryan A. Groves, Dominique G. Bihan, Thomas Rydzak, Ian A. Lewis

**Affiliations:** ^1^Department of Biological Sciences, University of Calgary, Calgary, AB, Canada; ^2^Department of Biomedical Engineering, University of Calgary, Calgary, AB, Canada

**Keywords:** metabolomics, antibiotic susceptibility testing (AST), metabolic boundary fluxes, liquid chromatography-mass spectrometry, metabolic preference assay, bacteria identification, fabrication

## Abstract

Metabolomics is a mainstream strategy for investigating microbial metabolism. One emerging application of metabolomics is the systematic quantification of metabolic boundary fluxes – the rates at which metabolites flow into and out of cultured cells. Metabolic boundary fluxes can capture complex metabolic phenotypes in a rapid assay, allow computational models to be built that predict the behavior of cultured organisms, and are an emerging strategy for clinical diagnostics. One advantage of quantifying metabolic boundary fluxes rather than intracellular metabolite levels is that it requires minimal sample processing. Whereas traditional intracellular analyses require a multi-step process involving extraction, centrifugation, and solvent exchange, boundary fluxes can be measured by simply analyzing the soluble components of the culture medium. To further simplify boundary flux analyses, we developed a custom 96-well sampling system—the Microbial Containment Device (MCD)—that allows water-soluble metabolites to diffuse from a microbial culture well into a bacteria-free analytical well via a semi-permeable membrane. The MCD was designed to be compatible with the autosamplers present in commercial liquid chromatography-mass spectrometry systems, allowing metabolic fluxes to be analyzed with minimal sample handling. Herein, we describe the design, evaluation, and performance testing of the MCD relative to traditional culture methods. We illustrate the utility of this platform, by quantifying the unique boundary fluxes of four bacterial species and demonstrate antibiotic-induced perturbations in their metabolic activity. We propose the use of the MCD for enabling single-step metabolomics sample preparation for microbial identification, antimicrobial susceptibility testing, and other metabolic boundary flux applications where traditional sample preparation methods are impractical.

## Introduction

Metabolomics is a mainstream strategy for understanding complex biological processes and mapping the molecular underpinnings of disease (Kell and Oliver, 2016; Pinu et al., 2019). Liquid chromatography-mass spectrometry (LC-MS) has emerged as the main platform for performing these analyses and increasing technological advances have allowed LC-MS to capture ever broader transects of the metabolome (Metz et al., 2007; Lu et al., 2017; Cui et al., 2018). Over the past several years, there has been an increasing emphasis on developing high-sensitivity LC-MS methods that capture the largest possible range of metabolites present in a given sample (Monge et al., 2019; Araujo-León et al., 2020). While these sensitivity-focused methods have enabled researchers to characterize astonishing chemical diversity in a wide range of organisms, these methods are difficult to implement in large-scale studies due to their use of long gradients and due to difficulties in conducting stable quantitative analyses over large-scale studies.

Recently, there has been increasing interest in establishing quantitatively robust metabolomics assays that can be applied to large-scale cohort studies (Zheng et al., 2018; Cao et al., 2020). One of the most promising approaches is tracking metabolic boundary fluxes—the rates at which metabolites are consumed or produced by *in vitro* cell cultures—to map intracellular metabolic activities (Orth et al., 2010; Pinu and Villas-Boas, 2017; Hui et al., 2020). Quantifying the rates that molecules are secreted or consumed can provide extensive insight into cellular function, the activity of metabolic networks (Liang et al., 2011; Hollinshead et al., 2014), and nutritional dependencies of diverse organisms (Orth et al., 2010). This emerging strategy is useful because it is quantitatively robust, can be applied to large-scale cohorts, and can rapidly capture phenotypic information about microbes, such as their species and antibiotic susceptibility profiles (Rydzak et al., 2022). In summary, measuring microbial boundary fluxes provides a robust mechanism for understanding the metabolism of cultured organisms. Notably, LC-MS methods that quantify these fluxes are a powerful emerging strategy in clinical diagnostics (Rydzak et al., 2022), improving biofuels production (Hollinshead et al., 2014; Bideaux et al., 2016), decoding microbiome interactions (Antoniewicz, 2020), understanding microbial community interactions (Khandelwal et al., 2013; Perez-Garcia et al., 2016), and enabling microbial engineering projects (Munro and Kell, 2021).

One of the primary advantages of studying microbes via their metabolic boundary fluxes is its simplicity. The metabolites that are consumed or secreted by *in vitro* cultures are generally water soluble, relatively abundant, and less chemically diverse than intracellular metabolites (Groves et al., 2022). Moreover, they are accessible via relatively simple sample preparation workflows that separate cells from the metabolites present in the culture medium (Pinu and Villas-Boas, 2017). These analyses could be further simplified if they were coupled to a device that physically separated the cells from the metabolite. To achieve this, we developed the Microbial Containment Device (MCD), a novel two-chamber sampling platform that separates microbial specimens in one chamber from a sterile analytical chamber via a semipermeable membrane. The primary goal of the MCD is to enable microbial boundary flux analyses via a single-step metabolomics sample processing method that minimizes human error, helps prevent sample cross-contamination, and minimizes technical error across large-scale cohorts. The MCD uses a similar design strategy to the commercial Transwell ^®^ (Corning) device, but it addresses two shortcomings of the Transwell ^®^ for use in high-throughput microbial metabolomics studies: (1) the pore size of the Transwell ^®^ (0.4 μm) allows some bacteria to pass through the membrane and (2) the high cost of the Transwell ^®^ is incompatible with the needs of large-scale metabolomics studies. To demonstrate the applicability of the MCD in replacing a traditional microbial culture workflow, we used the MCD to differentiate four bacteria species (*Escherichia coli, Klebsiella pneumoniae, Enterococcus faecalis*, and *Staphylococcus aureus*) and measure their antibiotic susceptibility profiles. Our data show that the MCD can (1) accurately reproduce traditional microbial culture workflows; (2) allow the user to analyze samples with no additional sample preparation steps once samples are loaded into the device; (3) facilitate pathogen identification (ID) and empirically measure antibiotic susceptibility profiles of these pathogens; and (4) maintain a sterile mass spectrometry analysis chamber. In summary, we introduce this tool as a simple device for enabling large-scale metabolomics projects where the emphasis is on quantifying metabolic boundary fluxes.

## Materials and methods

### Design principle of the microbial containment device

**FIGURE 1:**
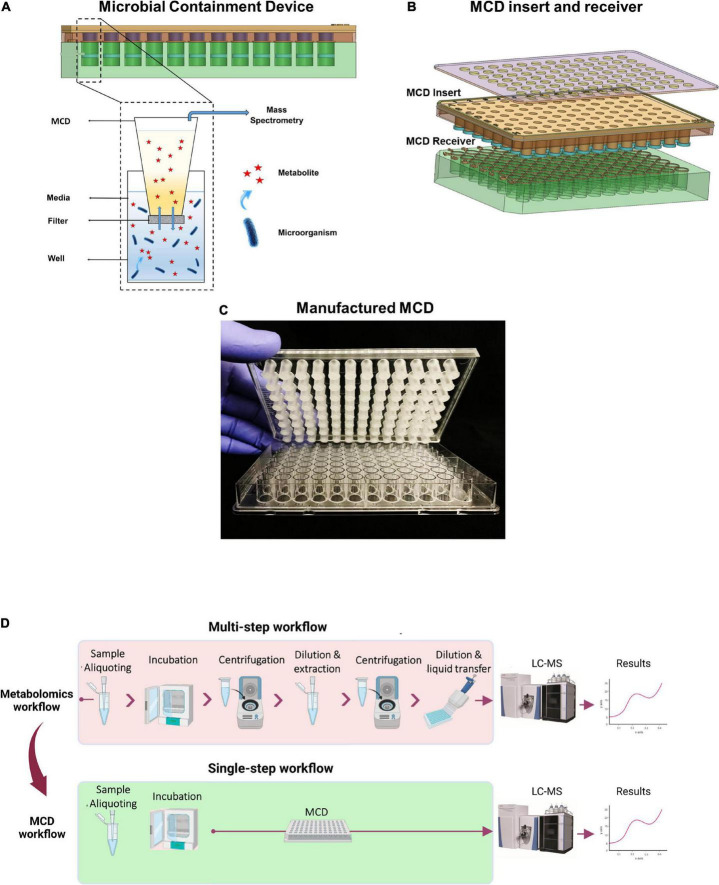
**(A)** Schematic view of the MCD design concept, **(B)** MCD insert and receiver, **(C)** assembled MCD, and **(D)** optimized MCD metabolomics workflow, versus conventional metabolomics sample preparation.

The MCD is a plastic insert that fits into a standard-format 96-well microplate. The MCD has 96 sampling inserts and the bottom of each is enclosed, with a 0.2-μm semi-permeable membrane. When the MCD is inserted into a 96-well microplate, the membrane creates a lower microbial growth chamber and upper analytical chamber. Media components, including small molecules such as metabolites, can freely diffuse through the membrane between the upper and the lower compartments (Figures 1A,B). The MCD is compatible with LC instrumentation that handles 96-well plates (e.g., Thermo Scientific™ Vanquish™ UHPLC system), and the MCD insert was designed to allow the Vanquish LC sampling needle to extract samples from the top of the MCD without piercing the membrane to enable simplified sample preparation for large cohort studies (Figure 1D).

### Fabrication of the microbial containment device

We used a rapid prototyping workflow to design, fabricate, and test the MCD. We used Fusion 360 software to design the MCD and performed 3D printing with high temp resin and a Form 3 SLA 3D printer (Formlabs, Boston, MA, United States). We then post-washed the 3D printed component in an isopropyl alcohol bath to dissolve liquid resin followed by post-curing using 405 nm light along with a heating system (based on the Formlabs manufacturing instructions for high temperature resin^1^). A Universal Laser Systems VLS3.60DT^2^ (Canadian Engravers Supply, Mississauga, ON, Canada) was used to cut the filter and double-sided tape for filter assembly. We used an Anprolene AN74j ethylene oxide gas sterilization machine (Andersen Sterilizers, Inc., Haw River, NC, United States) to sterilize the prototype MCD before testing and validation. After we performed proof-of-concept testing and validation of the device, we designed a custom mold for the injection molding and mass manufacturing processes. This required us to develop a new semi-automatic machine and assembly technique for polycarbonate filter (0.2 μm pore size) assembly in our lab. We also developed a Quality Control (QC) procedure (including visual testing, clinical testing, and validation) to ensure repeatability and reliability of the manufactured MCD. Commercial MCD platforms can be ordered from Fluidome, Inc.^3^ (Calgary, AB, Canada). All steps of development are shown in Supplementary Figure 1.

### Numerical simulation

To optimize the MCD design, we computationally modeled the metabolite diffusion and dynamic changes of metabolite concentration over time within the proposed device. We used COMSOL Multiphysics software^4^, a finite element simulation platform that spans all simulation steps from model design and geometries, selection of appropriate material and physics, solving, and postprocessing results, to simulate the diffusion rate in the MCD. For this study, we created a 3D model for one well where the bottom well geometry was 7 mm diameter × 4 mm height, and the top well geometry was 4 mm diameter × 4 mm height. We considered a filter membrane with 10% porosity on the interface of both top and bottom wells (porosity is the percent of the total surface area occupied by the pores) based on the polycarbonate filter membrane specification datasheet. For the boundary condition for this study, we assumed initial concentrations of 0 and 2 mM on the top and bottom wells, respectively (as 2 mM meets or exceeds the instrumental upper limit of quantification for most metabolites of interest). We considered the ambient pressure and temperature to be 100 kPa and 310 K, respectively, and water as the liquid material. Furthermore, we considered free tetrahedral mesh (extra fine), including 149,756 mesh elements, in this study. We used the transport of diluted species physics and a time-dependent study to monitor the concentration change over 6 h on the top and bottom of a single 3D well. The dynamic change of concentration was simulated in the 3D model over the cross-sectional surface and over the centerline of the well from bottom to top. Additionally, to ensure that the size of mesh and number of mesh elements had no impact on our result, we ran the mesh dependency test. The purpose of the mesh dependency test is to determine the correct mesh size (ranging from coarse to extra fine mesh) that leads to a consistent result (in this case, the diffusion rate of metabolites) in the simulation. This means that the simulation result remains independent from the mesh size. After running the simulation and gradually increasing the mesh density, we determined that free tetrahedral mesh (extra fine), including 149,756 mesh elements, produced the most consistent result for our simulation.

### Strains, growth, and sample preparation

Bacterial strains were grown on BD BBL™ Mueller Hinton II Broth (BD Biosciences; VWR, Edmonton, AB, Canada), prepared as per the manufacturer’s instructions. The Mueller Hinton broth was autoclaved at 121.5°C for 20 min (Primus Sterilizer, Omaha, NE, United States) and cooled to room temperature. The prepared medium was stored at 4°C. Mueller Hinton (MH) agar plates were prepared in the same way but with supplementation of 1% agar before autoclaving.

Different bacterial strains including *Escherichia coli* (*EC* 1, 2, 3, 4), *Klebsiella pneumoniae* (*KP* 1, 2, 3, 4), *Enterococcus faecalis* (*EF* 1, 2, 3), and *Staphylococcus aureus* (*SA* 1, 2, 3, 4) were purchased from the American Type Culture Collection (ATCC) or provided by the Center for Disease Control (CDC) (see Table 1).

**TABLE 1:** Organism names and strains used in this study.

Sample name	Organism name	Strain	Sample name	Organism name	Strain
*EC (1)*	*E. coli*	*ATCC 25922*	*KP (1)*	*K. pneumoniae*	*ATCC 700603*
*EC (2)*	*E. coli*	*BAA-196*	*KP (2)*	*K. pneumoniae*	*ATCC BAA-1705*
*EC (3)*	*E. coli*	*SAMN04014854*	*KP (3)*	*K. pneumoniae*	*SAMN04014953*
*EC (4)*	*E. coli*	*SAMN04014978*	*KP (4)*	*K. pneumoniae*	*SAMN04014954*
*SA (1)*	*S. aureus*	*ATCC 25923*	*EF (1)*	*E. faecalis*	*ATCC 29212*
*SA (2)*	*S. aureus*	*ATCC 43300*	*EF (2)*	*E. faecalis*	*ATCC 51299*
*SA (3)*	*S. aureus*	*ATCC BAA-976*	*EF (3)*	*E. faecalis*	*ATCC 19433*
*SA (4)*	*S. aureus*	*ATCC BAA-977*			

Bacteria were incubated overnight (36.5°C, 5% CO_2_ and 17.5% O_2_) on Mueller Hinton (MH) agar plates prior to experiments, and 17.5% O_2_. All experiments were performed in the Thermo 1,300 Series A2 biosafety cabinet. To create initial stock solutions, we picked a bacterial colony from an agar plate and diluted it in 4 mL saline to reach 0.5 McFarland (∼7.5E6 CFU/mL). Optical density values at 600 nm (OD_600_) were measured in a microplate reader (Thermo Scientific™ Multiskan™ GO).

### Experimental workflow

Antibiotic panels containing cefazolin (CFZ; 2, 8 μM), gentamicin (GEN; 4, 8 μM), ampicillin (AMP; 8, 32 μM), ciprofloxacin (CIP; 1, 4 μM), ceftriaxone (CRO; 1, 2 μM), and meropenem (MER; 1, 2, 4, 8 μM) at Clinical and Laboratory Standards Institute (CLSI) breakpoint concentrations were made by adding the antibiotic solution to each well of a 96-well plate and drying the plates for 4 h in the biosafety cabinet.

#### Conventional metabolomics workflow

270 μl MH medium was added to each well of the MCD receiver (96-well plate). Wells were inoculated using 30 μl of bacterial suspension (0.5 McFarland). The plate was sealed using a gas permeable sealer (rayon, 139.7 μm pore size; VWR) and incubated at 36.5°C, 5% CO_2_ and 17.5% O_2_. After 4 h, a 10 μl aliquot from each well was diluted with 90 μl 50% methanol (Thermo Scientific™ Optima™ LC/MS grade reagent)/50% water (v/v; Optima LC/MS grade reagent) in a 96-well PCR plate (VWR 96-well Real-Time PCR skirted plate). The plate was centrifuged at 4,000 rpm for 10 min (Thermo Scientific™ Sorvall™ Legend™ XTR centrifuge). Subsequently, 70 μl of the supernatant was transferred to a new 96-well sampling plate (Greiner Bio-One Masterblock, 0.5 mL V-bottom, sterile; Monroe, NC, United States) and diluted with 70 μl 50% methanol for a total sample dilution of 1:20 from the starting concentration. The samples were analyzed via LC-MS.

#### Microbial containment device workflow

The following workflow was used to compare the results from the MCD to the conventional metabolomics workflow. However, the MCD is designed to be used with the Thermo Scientific™ Vanquish™ Charger Module to enable the optimized workflow, as shown in Figure 1D. 220 μl MH medium was added to each well of the MCD receiver (96-well plate). Wells were inoculated using 30 μl of bacterial suspension (0.5 McFarland). The MCD insert was placed into the MCD receiver followed by transferring 50 μl MH medium to each MCD insert (Figure 1). The MCD was then sealed using a gas permeable sealer (rayon, 139.7 μm pore size; VWR). Samples from the MCD were then incubated, extracted, and analyzed following the conditions described above.

### Metabolomics analysis and data visualization

#### Ultra-high-performance liquid chromatography-mass spectrometry

All metabolomics testing and characterization steps were carried out in the Calgary Metabolomics Research Facility (CMRF). Our methods have been described elsewhere (Groves et al., 2022; Rydzak et al., 2022). Briefly, we used a Vanquish UHPLC platform coupled to a Thermo Scientific™ Q Exactive™ HF Hybrid Quadrupole-Orbitrap™ mass spectrometer. Metabolites were resolved via hydrophilic interaction liquid chromatography (HILIC; 100 mm × 2.1 mm Thermo Scientific™ Syncronis™ LC column 2.1 μm particle size). A 600 μl/min binary gradient consisting of 20 mM ammonium formate at pH 3.0 in LC-MS grade water (Solvent A; Optima LC/MS reagent) and 0.1% formic acid (% v/v) in LC-MS grade acetonitrile (Solvent B; Optima LC/MS reagent) was used as a mobile phase. Metabolites were eluted using a 15-min gradient. Samples were ionized via electrospray ionization (positive mode for tyramine and negative mode for all other metabolites) with the auxiliary gas of 15 (arbitrary units), sweep gas of 2 (arbitrary units), sheath gas of 35 (arbitrary units), auxiliary gas temperature of 300^°^C, spray voltage of −2000 V, and capillary temperature of 275^°^C. Data were acquired in full scan mode (50–750 *m/z*) with a 240,000 resolving power, an automatic gain control target of 3e^6^, and a maximum injection time of 200 ms. All standards were purchased from Sigma-Aldrich (CAS Numbers: Mevalonate—1255502-07-8; Succinate—6106-21-4; Tyramine—51-67-2; Urocanate—104-98-3) and used to verify the biomarkers described in Rydzak et al. (2022). Raw MS data were converted to mzXML file format using MSConvert GUI software (Chambers et al., 2012) and analyzed using MAVEN (El-MAVEN v0.12.0) (Melamud et al., 2010).

### Statistical analysis

All experiments were replicated with three biological replicates and three technical replicates for each treatment and we used GraphPad Prism 9 software (Skinner, 2018) to calculate the statistical differences between treatments using one-way ANOVA with *post hoc* Tukey correction, where *p < 0.05; ^**^p < 0.01; ^***^p < 0.001; and ^****^p < 0.0001. All the statistical methods for finding microbial biomarkers were reported in our previous study (Rydzak et al., 2022).

## Results

### Diffusion characterization (empirical testing)

**FIGURE 2:**
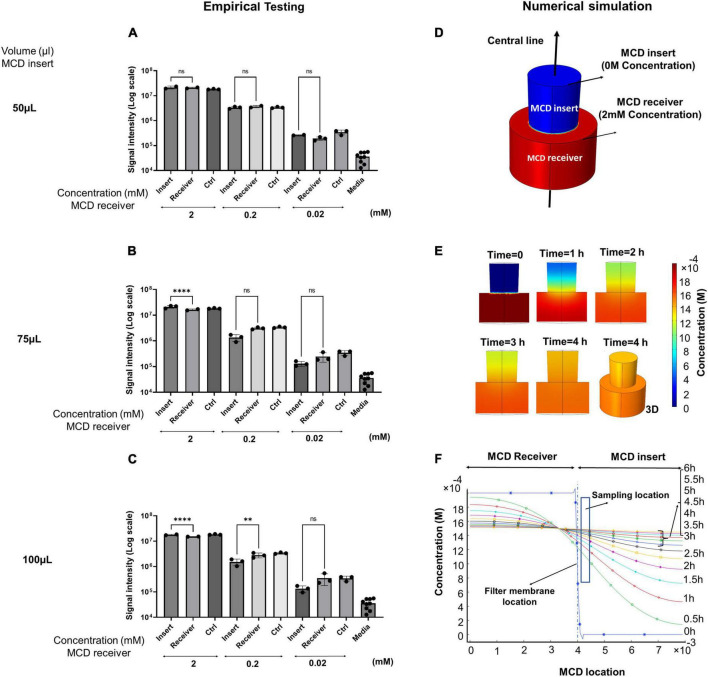
**(A–C)** Empirical versus **(D–F)** simulated diffusion kinetics of succinate dissolved in water in the MCD device. Succinate was added to the MCD receiver (bottom well) and the effects of initial succinate concentration (2, 0.2, and 0.02 mM) and insert aqueous volume (upper well) (50, 75, and 100 μl) on the final concentrations of succinate observed after 4 h at 37°C. Succinate signal intensities for MCD insert versus receiver **(A)** 50 μl insert aqueous volume, **(B)** 75 μl insert aqueous volume, and **(C)** 100 μl insert aqueous volume. **(D)** A 3D model of metabolite concentrations in the MCD was constructed and **(E)** metabolite diffusion was computed over a 4-h incubation. **(F)** Metabolite concentrations observed across a vertical transect of sampling points in the MCD crossing from the bottom of the receiver, through the membrane, and to the top of the insert are shown across a time point ranging from 0 to 6 h. For bar graphs, ^**^p < 0.01 and ^****^p < 0.0001.

The primary function of the MCD is to separate microbes in the lower growth chamber from the upper analytical chamber but allows microbial metabolites to freely diffuse between the two chambers. To assess the efficacy of the prototyped MCD, we conducted a series of diffusion assays and quantified the kinetics of diffusion between lower and upper wells. Two parameters affecting diffusion equilibrium timelines were analyzed: (1) the volume of liquid in the MCD insert and (2) the concentration of metabolites in the MCD receiver. We used succinate as a representative small molecule metabolite for this study since its size, cross-sectional area, and chemical properties are broadly representative of soluble metabolites found in microbial growth media; 250 μl of succinate solutions at varying concentrations (2, 0.2, and 0.02 mM) was introduced into the MCD receiver (bottom well). Then, 50, 75, or 100 μl of water was added to the MCD insert (top well). Samples were incubated for 4 h and aliquots were then taken from each MCD insert, diluted 20-fold with 50% methanol, and succinate concentrations were measured by UHPLC-MS (Figures 2A–C). The results demonstrated that succinate freely diffused from the bottom to top wells. As expected, larger volumes in the top well slowed equilibrium timelines, whereas increasing metabolite concentration in the bottom wells accelerated diffusion. When succinate was prepared at a 2 mM concentration in the bottom well, we observed ∼10X faster diffusion than comparable wells prepared at 0.2 mM. These data demonstrated that our prototype enabled the diffusion of metabolites between the upper and lower wells and that diffusion rates followed the expected behaviors.

### Diffusion characterization (numerical simulation)

A critical consideration in conducting metabolomics experiments in the MCD is the time required for a microbe to reach equilibrium between the lower and upper wells. We used COMSOL Multiphysics software to conduct a numerical simulation of diffusion and to quantify the diffusion rate between 0 and 6 h for molecules dissolved in liquids in an aqueous (water) solution. Since samples were taken within a 1 mm distance from the filter membrane of the MCD insert during empirical testing, we considered this same sampling zone for evaluation of the diffusion rate in the MCD for the numerical simulation. The 3D schematic showing the diffusion of metabolites in one well including the MCD receiver and insert along the centerline is shown in Figure 2D. After running the 2D and 3D simulations, the gradient of concentration over the cross-sectional surface of the model for 0, 1, 2, 3, and 4 h are shown in Figure 2E. The gradient of concentration for the 3D model at 4 h is also shown in Figure 2E. Figure 2F illustrates the concentration change for metabolites in both the MCD receiver and insert over the center line from 0 to 6 h. These simulations showed that both the MCD insert and receiver reach equilibrium conditions within 4 h in the sampling zone, which is in agreement with empirical testing using 2 mM succinate in the bottom well and 50 μl water in the upper. Based on these simulations, we determined that sampling time points of 4 h or longer are sufficient for high-abundance metabolites to reach equilibrium.

### Sterility testing

**FIGURE 3:**
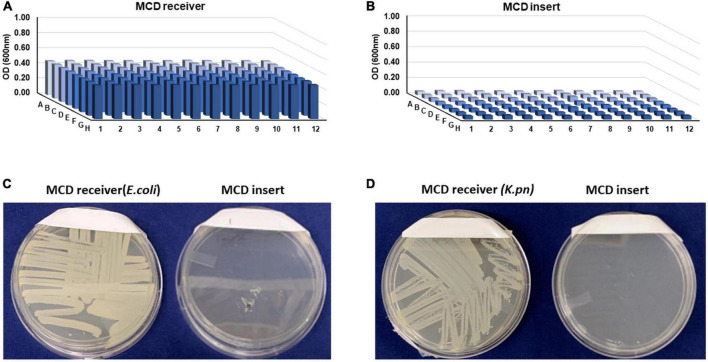
Sterility testing of the MCD insert (upper, analytical chamber) following an 8-h incubation of the MCD receiver seeded with bacteria. Bacterial cell densities (OD_600_) in the MCD receiver **(A)** and MCD insert **(B)** show that growth only took place in the receiver and not the insert. To further validate sterility of the insert, the MCD receiver and insert samples were streaked on agar plates and incubated overnight **(C,D)**.

One critical function of the MCD is to grow microbes in the lower chamber to ensure that they cannot traverse into the upper chamber where they could potentially contaminate the LC-MS hardware. To evaluate the efficacy of our membrane in separating the growth and the analytical chambers, we conducted a series of contamination (bottom-to-top) sterility tests using *E. coli*. For bottom-to-top sterility testing, 250 μl of MH medium containing either *E. coli* or *K. pneumoniae* (OD_600_ 0.01) was loaded into the MCD receiver. The MCD insert was placed on top of the MCD receiver and 100 μl of MH broth was added to each well of the MCD insert. Optical density measurements after 8 h on the MCD receiver were much higher than the MCD insert (Figures 3A,B, respectively) suggesting that it effectively contained the bacteria in the MCD receiver. To further ensure sterility of the analytical chamber (upper well), samples were streaked on agar plates and incubated overnight. No growth was observed on agar plates streaked with inoculum from the upper analytical chamber (Figures 3C,D), demonstrating that the MCD membrane successfully separated the microbial chamber (MCD receiver) and the analytical chamber (MCD insert). This analysis was repeated using both *S. aureus* and *E. faecalis*, and no bacterial growth was observed in the upper analytical chamber (Supplementary Figure 2).

### Species identification using metabolites

**FIGURE 4:**
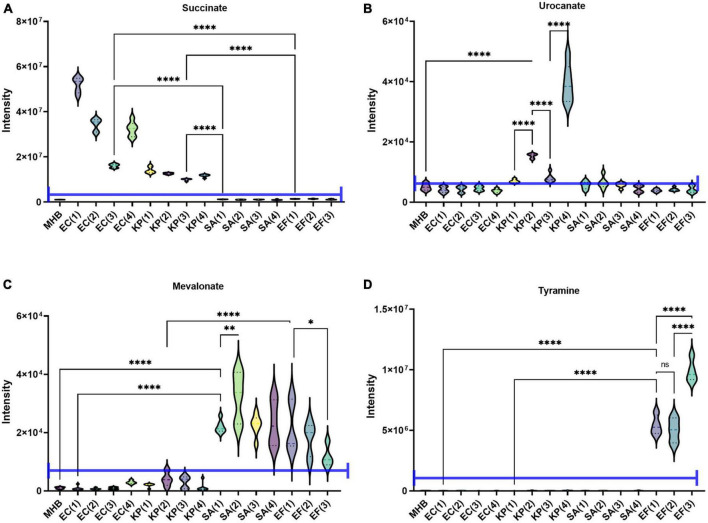
Demonstration of the MCD as a platform for identifying microbes *via* their metabolic boundary fluxes. *Escherichia coli* (EC 1, 2, 3, 4), *Klebsiella pneumoniae* (KP 1, 2, 3, 4), *Enterococcus faecalis* (EF 1, 2, 3), and *Staphylococcus aureus* (SA 1, 2, 3, 4) were identified based on the intensity of **(A)** succinate, **(B)** urocanate, **(C)** mevalonate, and **(D)** tyramine signals observed in the MCD insert following a 4 h incubation. The purple line in each plot shows the signal intensity threshold for each biomarker used to distinguish the four species. *p < 0.05; ^**^p < 0.01; and ^****^p < 0.0001.

**FIGURE 5:**
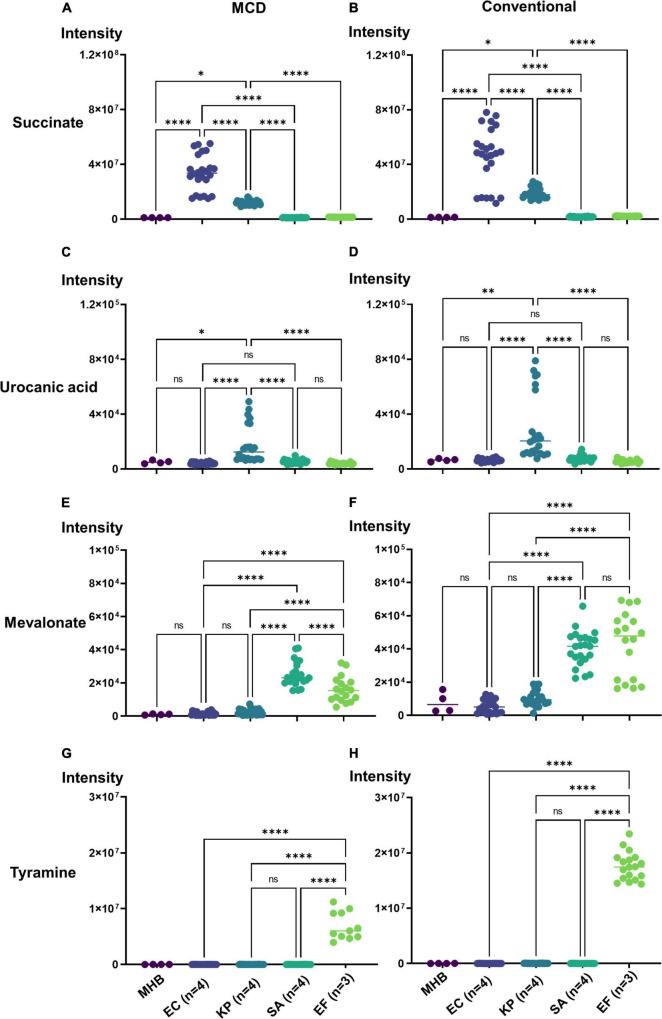
Bacteria identification using the MCD **(A,C,E,G)** versus the a conventional metabolomics workflow **(B,D,F,H)** for EC (*n* = 4), KP (*n* = 4), EF (*n* = 3), and SA (*n* = 4), strains. For all plots, *p < 0.05; ^**^p < 0.01; and ^****^p < 0.0001.

One obvious application of the MCD is in quantifying microbial boundary fluxes, which were recently shown to be an effective strategy for differentiating bloodstream pathogens (Rydzak et al., 2022). To illustrate the utility of the MCD, we inoculated MCD plates with four bloodstream pathogens (*E. coli*, *K. pneumoniae*, *E. faecalis*, and *S. aureus;* three to four unique isolates per species) and analyzed the analytical chamber for the presence of diagnostic biomarkers following a 4-h incubation. It has been reported previously (Rydzak et al., 2022) that both *E. coli* and *K. pneumoniae* produce succinate, but *K. pneumoniae* also produces urocanate. Similarly, both *S. aureus* and *E. faecalis* produce mevalonate, but *E. faecalis* also produces tyramine. Thus, quantifying succinate, urocanate, mevalonate, and tyramine are sufficient for differentiating these species. As anticipated, microbes incubated in the MCD were differentiable based on these biomarker patterns. Significant succinate production was observed in *E. coli* and *K. pneumoniae* cultures (Figure 4A), and urocanate production was uniquely observed in the *K. pneumoniae* cultures (Figure 4B). Similarly, we observed significant mevalonate production in *S. aureus* and *E. faecalis* cultures (Figure 4C), but tyramine production was only observed in *E. faecalis* cultures (Figure 4D). Furthermore, biomarker intensities observed in the MCD were comparable to those observed via the conventional metabolomics method (Figure 5). In summary, the MCD enables boundary flux analyses and microbes grown in the MCD produce diagnostic markers at levels that are comparable to those observed via conventional sample preparation methods.

### Antibiotic susceptibility testing

**FIGURE 6:**
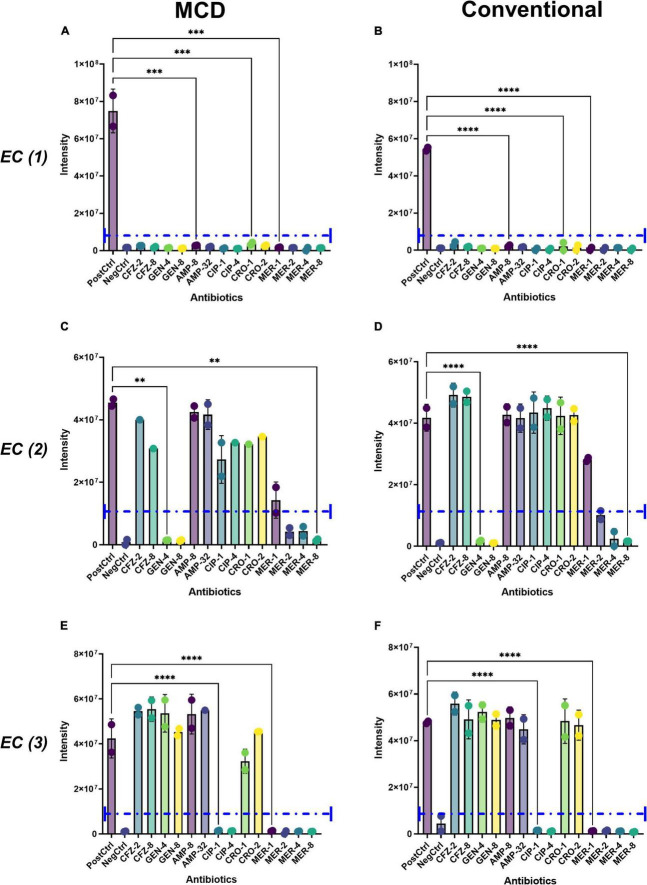
Demonstration of the MCD as a platform for antibiotic susceptibility testing. The metabolic activity of *E. coli* (EC, 1, 2, 3) was analyzed when cells were grown in a panel of antibiotics including cefazolin (CFZ 2, 8 μM), gentamicin (GEN 4, 8 μM), ampicillin (AMP 8, 32 μM), ciprofloxacin (CIP 1, 4 μM), ceftriaxone (CRO 1, 2 μM), and meropenem (MER 1, 2, 4, 8 μM). Boundary fluxes were quantified *via* LC-MS using both the **(A,C,E)** MCD and **(B,D,F)** conventional metabolomics workflows. The purple dotted line in each plot shows the signal intensity threshold considered as biomarker production. For all plots, ^**^p < 0.01, ^***^p < 0.001, and ^****^p < 0.0001.

Recently, Rydzak et al. (2022) showed that antibiotic-sensitive microbes have significant perturbations in their metabolic boundary fluxes when exposed to antibiotics, whereas antimicrobial resistant strains have a minimal metabolic response to antibiotic exposure. They showed that these perturbations can be used to determine the minimal inhibitory concentrations of antibiotics. To assess the applicability of the MCD for antibiotic susceptibility testing (AST), *E. coli* strains (*n* = 3; each with a distinct susceptibility profile as described in Supplementary Figure 3) were incubated with a panel of antibiotics containing cefazolin (CFZ), gentamycin (GEN), ampicillin (AMP), ciprofloxacin (CIP), and meropenem (MER) at CLSI breakpoint concentrations as described in the section “Materials and methods.” The sensitivity profiles of each strain were known based on their ATCC strain information (Supplementary Figure 4). These phenotypes were verified using the MCD by measuring optical density (OD_600_) after a 4-h incubation in our antibiotic panel (Supplementary Figure 4). As expected, the MCD-based growth matched the patterns expected based on the ATCC literature for each strain (Figure 6). Antibiotic-induced perturbations in the metabolic activity of each microbe were then assessed using both the MCD and conventional metabolomics methods. LC-MS analyses showed that antibiotic exposure inhibited succinate production in antibiotic-sensitive strains but not in antibiotic-resistant strains. Moreover, the pattern of inhibition followed the known resistance profiles for each strain. Furthermore, the overall intensity of succinate signals observed *via* the MCD corresponded to the patterns observed *via* the conventional metabolomics workflow (Figure 6). In summary, MCD-based analyses of boundary fluxes enable antibiotic susceptibility profiles to be determined.

## Discussion

In this study, we showed that a simple consumable device, the MCD, enabled us to characterize the metabolic boundary fluxes of microbial cultures via a streamlined sample preparation procedure. We demonstrated that: (1) metabolites produced by microbes diffused freely through the MCD membrane over a 4 h incubation; (2) the membrane maintains the sterility of upper wells in the device; and (3) this tool can be used to both identify microbial species and empirically measure antibiotic susceptibility profiles. In summary, this study shows that the MCD functions as a tool for single-step metabolomics experiments and *in vitro* metabolic assays.

Although we illustrated the use of this tool in the context of microbial diagnostics, this concept is broadly applicable to any metabolomics study where the objective is to quantify the metabolic boundary fluxes of *in vitro* cell cultures. This wider range of applications includes biofuel production studies, such as for quantifying butyric acid and other products of fermentation by *Clostridium acetobutylicum* (Jang et al., 2013; Liu et al., 2019) and for studying the microbial bioconversion of plant polymers such as pectin and lignocellulose to biofuels (Kuivanen et al., 2019; Lu et al., 2022). The MCD could also provide a useful vehicle for systematically screening large gene knockout libraries in microbial engineering projects (Porokhin et al., 2021). Although the MCD was evaluated here in the context of bacterial metabolism, we anticipate it could be readily adapted to studying mammalian cell culture models (Allen et al., 2003; Zukunft et al., 2018; Lagziel et al., 2019; Wright Muelas et al., 2020), biomarker discovery (Tolstikov et al., 2020), pharmaceutical lead screening (Tomita et al., 2018), environmental monitoring (Lankadurai et al., 2013), microbiology (Ye et al., 2022), plant biology (Kumar et al., 2017), and food chemistry (Cevallos-Cevallos et al., 2009).

### Limitations

Although the MCD may simplify metabolomics studies for a wide range of studies, it has some obvious limitations that warrant consideration. One important point is that the diffusion rates of metabolites limit the minimum sampling times possible on this platform. Our data show that small water-soluble metabolites take ∼4 h to reach equilibrium, which precludes the use of the MCD in studying transient phenotypes. In addition, diffusion rates are directly affected by molecular size. Although larger molecules (e.g., lipoproteins, albumin, enzymes, and antibodies) may freely pass through the MCD filter, the longer incubation times needed to equilibrate these large molecules (Nikaido and Rosenberg, 1981) may make them unsuitable for study in the MCD. This will be particularly problematic for high flux biological model systems (e.g., quickly dividing cells that are grown at high density). Gas availability in the microbial growth chamber may also be problematic in high flux systems. Although microbial growth rates observed with and without the MCD receiver suggest O_2_/CO_2_ availability was not a growth-limiting factor in this study, the semi-permeable membrane used in the MCD will slow gas diffusion to the lower well and may affect some model systems. Another consideration that may affect the use of the MCD is the suspension state of the cells. The experiments herein illustrate diffusion times linked to uniformly suspended cells; adherent or sedimented cells will have longer equilibrium timelines since cellular metabolism will be concentrated at the bottom of the MCD receiver.

In summary, we introduce here a simple consumable device that enables metabolic boundary fluxes to be studied via a streamlined sample handling procedure. We illustrate the utility of the MCD platform for microbial identification and antibiotic susceptibility testing and propose its applicability to a wide range of studies.

## Data availability statement

All of the data used in this article are available on request from the authors.

## Author contributions

MM, TR, and IAL designed the experiments. MM designed, manufactured, and assembled the MCD and conducted the microbial growth, identification, and antibiotic susceptibility testing assays. MM, RA, and SL characterized the MCD. MM, RAG, and DGB collected mass spectrometry data. MM, SLB, TR, and IAL wrote the manuscript. All authors have read and approved the final version of the manuscript.

## Footnotes

1
www.formlabs.com

2
www.ulsinc.com

3
www.fluidome.ca

4
www.comsol.com
