# Supplementary material for: Microbial containment device: A platform for comprehensive analysis of microbial metabolism without sample preparation
Source: Front Microbiol. 2022 Sep 13;13:958785. doi: 10.3389/fmicb.2022.958785 (PMC9513318; doi:10.3389/fmicb.2022.958785)
Supplement: Supplementary file 1 [file Table_1.docx]

**Supplementary information:**

## **Microbial Containment Device (MCD): a platform for comprehensive analysis of microbial metabolism without sample preparation**

Mehdi Mohammadi^1,2^, Stephanie L. Bishop^1^, Raied Aburashed^2^, Saad Luqman^2^, Ryan A. Groves^1^, Dominique G. Bihan^1^, Thomas Rydzak^1^, Ian A. Lewis^1^*

The first stages of the development included rapid prototyping with a 3D printed version of the MCD, where Pressure-sensitive adhesive (PSA) was used to bond a 0.2 micron Polycarbonate filters to the insert wells and experiments were run with this configuration to validate the design. A low-cost production run was done with the first iteration of this device, by making a mold and injection molding some plates, upon which validation tests were run to test performance and efficacy.

The microbial containment device (MCD) is an integral part of the workflow, allowing for the incubation and sampling of the patient samples in one step. It is an insert meant to go into a 96-well microplate, with a permeable membrane at the bottom of each insert well that allows for the transfer of metabolites from the bacteria on the bottom to the sampling media on the top.


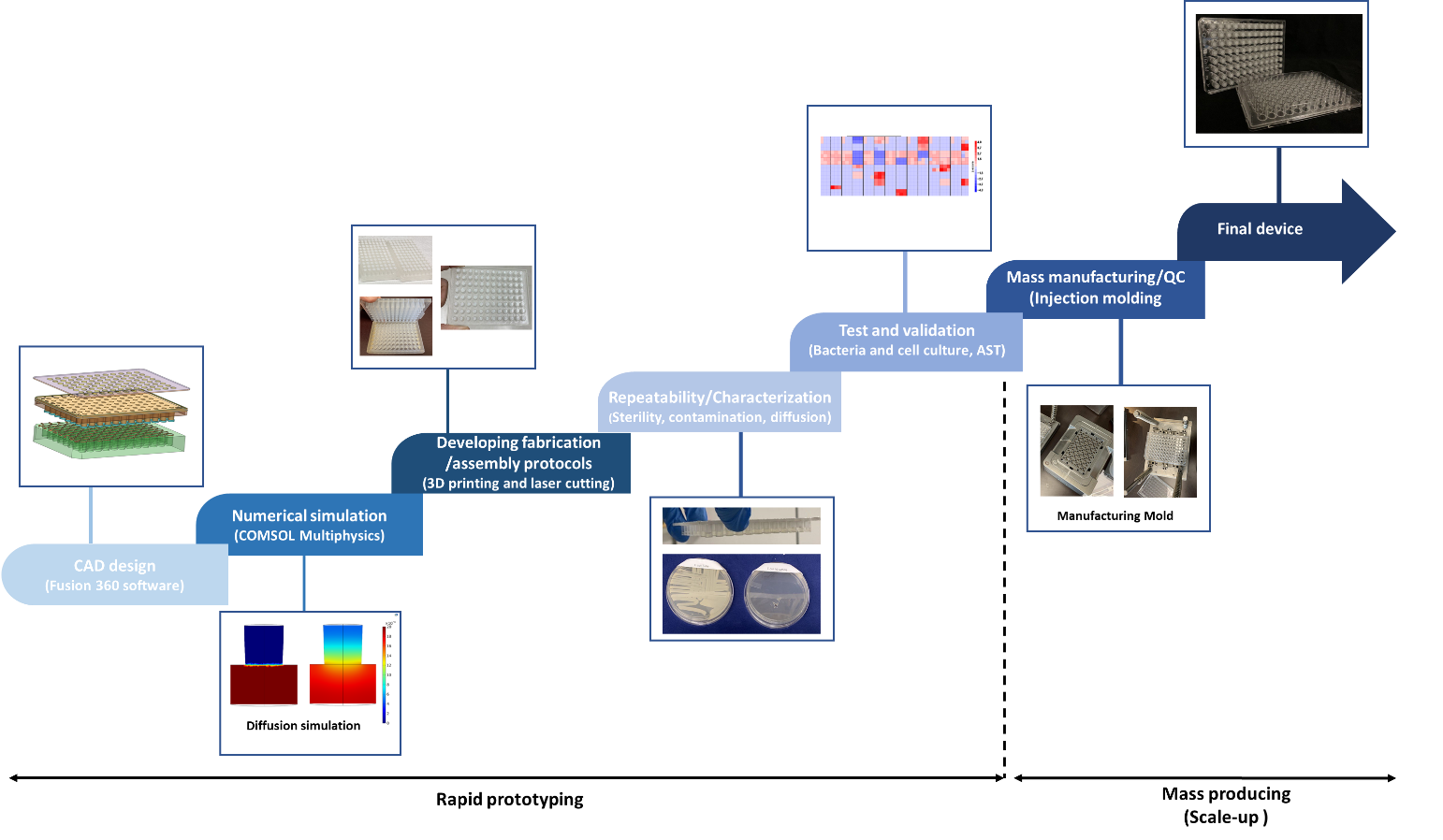


**Figure SI 1.** The MCD development steps include designing, simulation, fabrication, repeatability and validation, mass manufacturing (injection molding) and quality control.


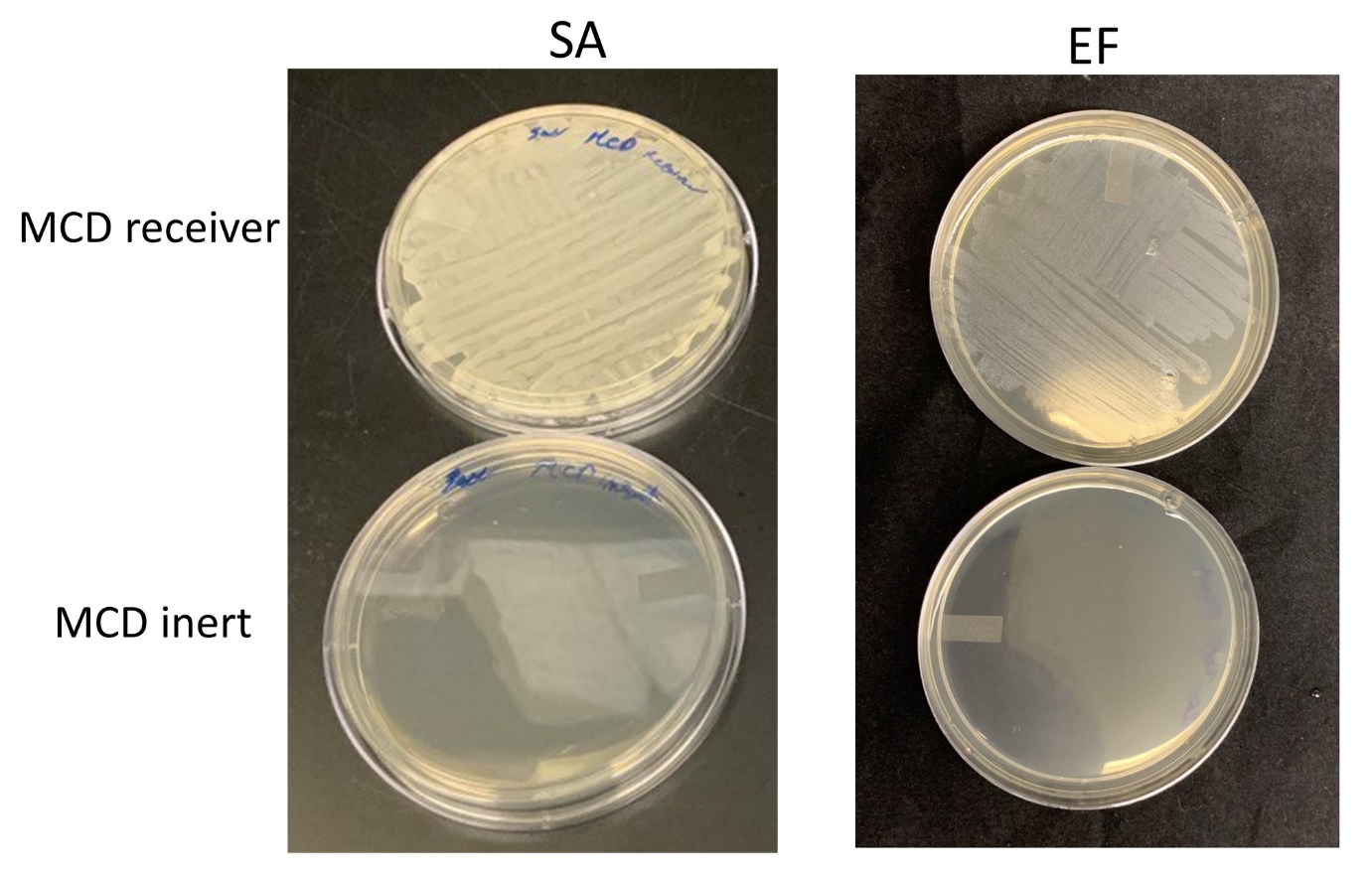


**Figure SI 2.** Sterility test for SA and EF.

We utilized different strains of *E. coli* (EC 1,2,3) bacteria with different resistance profiles for antibiotic susceptibility testing.

|  | CFZ | GEN | AMP | CIP | CRO | MER |
| --- | --- | --- | --- | --- | --- | --- |
| EC1 | I4 | S1 | S4 | S0.06 | S0.25 | S0.25 |
| EC2 | R32 | I8 | R32 | R8 | R32 | R4 |
| EC3 | R8 | R>16 | R32 | S0.25 | R32 | S0.12 |
|  |  |  |  |  |  |  |
|  |  | Resistant | Intermediate | Sensitive |  |  |

**Figure SI 3.** *E. coli* strains and resistance profiles.


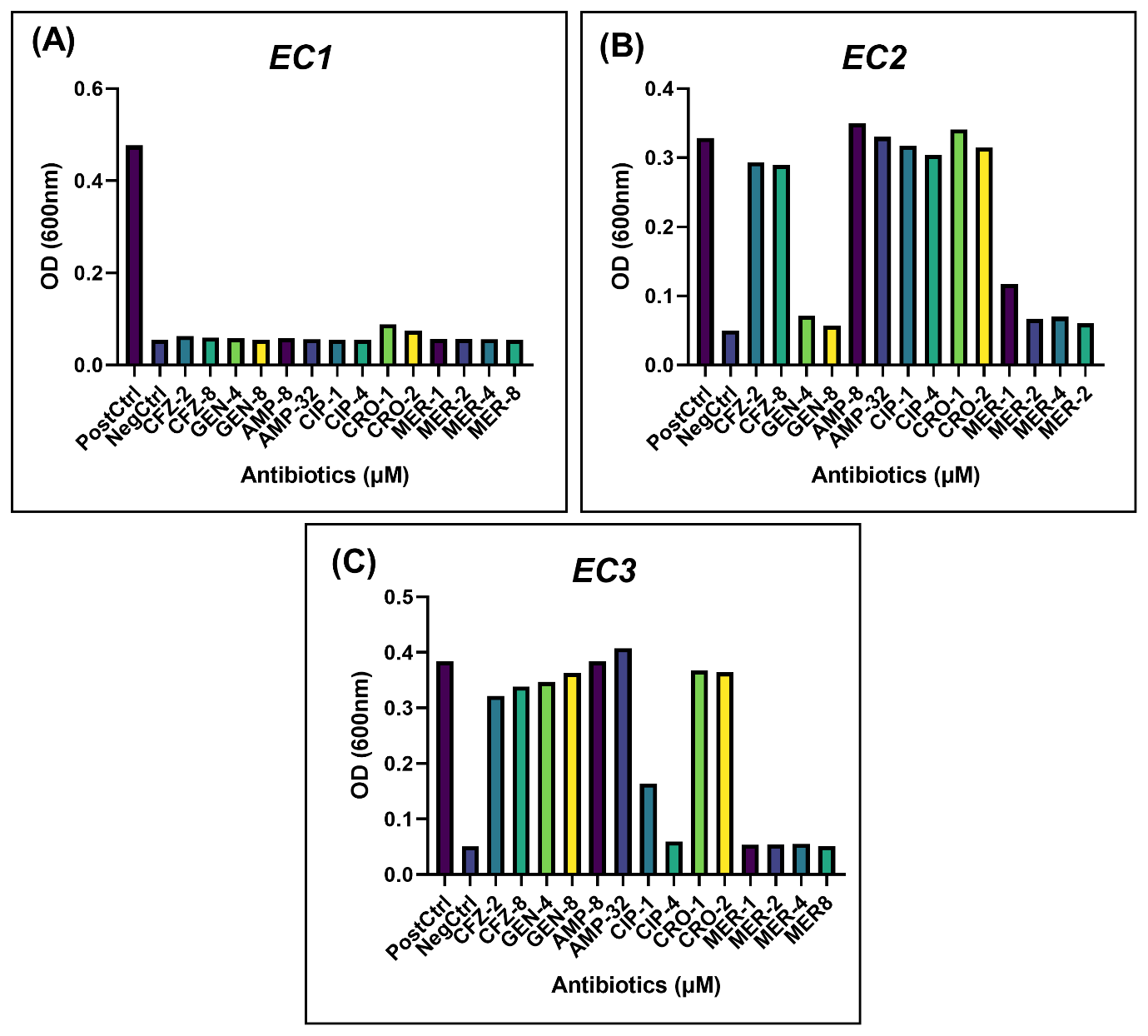


**Figure SI 4.** Bacterial optical density measurements for antibiotic susceptibility testing in the MCD. (a) EC1, (b) EC2, and (c) EC3.
